# Incidentally Found Rectal Carcinoid Tumor in a 46-Year-Old Female: The Potential for Complications and the Importance of Screening Guidelines

**DOI:** 10.7759/cureus.55163

**Published:** 2024-02-28

**Authors:** Rebecca Lapides, Akash Shah, Shubhneet Bal

**Affiliations:** 1 Department of Internal Medicine, The Robert Larner, M.D. College of Medicine, University of Vermont, Burlington, USA; 2 Department of Internal Medicine, Nuvance Health, Brookfield, USA; 3 Department of Pathology, Danbury Hospital, Danbury, USA

**Keywords:** colorectal screening and early detection, management of diabetes, management of hypertension, screening guidelines, gastrointestinal carcinoid tumor, rectal carcinoid tumors

## Abstract

Carcinoid tumors are rare neuroendocrine tumors that can be found in the gastrointestinal tract as well as other areas throughout the body. The neurosecretory nature of these tumors can have implications for other chronic diseases that patients may have, such as diabetes. Certain treatments that may be implemented for patients who have carcinoid tumors, such as somatostatin analogs and Everolimus, can also alter blood glucose control. This highlights the importance of diagnosing and treating carcinoid tumors as early as possible to avoid complications associated with metastasis and more intense treatment. With more advanced diseases, clinicians should consider the possible effects of carcinoid tumors and their treatments on other chronic conditions as they manage the patient. For gastrointestinal carcinoid tumors, colonoscopy screening guidelines are incredibly important to counsel patients on, as resection can yield a complete cure for carcinoid tumors when they are found at an early stage. Here, we describe the case of an incidentally diagnosed rectal carcinoid tumor in a 46-year-old female patient with a history of type 2 diabetes mellitus and hypertension.

## Introduction

Carcinoid tumors are rare neoplasms of neuroendocrine cell origin. They are capable of secreting serotonin into the systemic circulation, resulting in what is classically known as carcinoid syndrome. Carcinoid syndrome is characterized by symptoms including diarrhea, episodic flushing, bronchoconstriction, and eventual right-sided heart failure. Carcinoid tumors are also capable of secreting other neuroendocrine substances, including, but not limited to, dopamine, histamine, atrial natriuretic peptide, chromogranins, vasoactive intestinal polypeptide, somatostatin, and prostaglandins [1].

The neurosecretory nature of carcinoid tumors has implications in the management of other chronic conditions, especially with advanced disease, which must be considered when managing these patients. The ideal scenario would be that the disease is caught when surgical resection can yield a complete cure. However, the treatment protocols for more advanced tumors, specifically rectal carcinoid tumors, have not been formally established. Carcinoid tumors themselves, as well as certain existing treatments, can impact the management of other conditions, such as diabetes, by impacting glycemic control and balance [2]. Given that the incidences of neuroendocrine tumors, diabetes, and hypertension are rising [3,4], better elucidating the impact of carcinoid tumors and their treatments on such conditions, as well as formally establishing treatment protocols to optimize patient counseling is important. Patients also should be regularly counseled on screening recommendations that may enable earlier diagnosis. Here, we describe the case of a 46-year-old female patient, with pre-existing type 2 diabetes mellitus and hypertension, who was incidentally diagnosed with a rectal carcinoid tumor. This case highlights the benefits of following screening guidelines by discussing the potential future complications of this patient's condition had she opted out of the screening, as well as the importance of adequate patient counseling by the clinician so patients can make informed decisions, as this patient had initially expressed reluctance to undergo colonoscopy prior to receiving such counseling.

## Case presentation

The patient is a 46-year-old female with a past medical history of type 2 diabetes mellitus, hypertension, microcytic anemia, and menorrhagia who recently had liposuction and a gluteal augmentation. The patient had these procedures performed in another state, prior to establishing care in Connecticut with Nuvance Health. After the procedures, she was hospitalized for one night for observation due to hemoglobin dropping from 9.4g/dL to 8.7g/dL but ultimately did not require a blood transfusion. Several days later, she presented to the hospital complaining of generalized weakness and shortness of breath and was admitted to the hospital with a diagnosis of acute blood loss anemia. Initial laboratory work revealed microcytic anemia and the patient received two packed red blood cell transfusions. A contrast-enhanced CT scan of the abdomen and pelvis showed inflammation in the gluteal area with no discrete drainable fluid collection. The white blood cell count was elevated at 27 cells/µL and the patient was febrile. The patient was treated with a one-time 1g dose of vancomycin, ceftriaxone 2g daily for three days, anda one-time 500mg dose of meropenem, and then the meropenem was discontinued. After the initiation of antibiotic therapy, the leukocytosis began trending downward and the patient reported less pain. A CT scan was repeated three days later, which demonstrated an ill-defined fluid collection in the right gluteal muscle, which had mildly decreased in size, and no organized fluid collection to suggest an abscess. The patient was seen by interventional radiology, and it was determined that no fluid pocket was accessible for drainage. She continued to improve clinically and after she remained afebrile for 36 hours, she was discharged on oral doxycycline 100mg twice daily for seven days for treatment of post-surgical cellulitis. High-quality images of the above CT scans were not available to us, as the information was relayed via the patient's discharge paperwork when she established care in Connecticut.

The patient was seen in the office as an outpatient and continued to complain of pain at the surgical site, weakness, lightheadedness, and dizziness. On CBC, the patient’s white blood cell count was 12.8 cells/µL with a left shift and hemoglobin was low at 9.9g/dL. A referral was placed to infectious disease at this time. However, two days before the appointment, the patient reported to the hospital due to fatigue and concern for anemia. At this time, CBC showed that the white blood cell count had normalized to 7.9 cells/µL and the hemoglobin had risen to 10.7g/dL. The patient was reassured and discharged and saw infectious disease on the scheduled date, where it was determined that the patient was progressing as expected and the patient was counseled to call the office immediately if she developed worsening pain, erythema, inflammatory changes, malaise, or fever.

Approximately two weeks later, the patient’s hemoglobin had normalized to 12.2g/dL; however, due to her history of chronic microcytic anemia, she was referred to hematology to evaluate for possible underlying thalassemia. The patient stated that she has been anemic all her life secondary to uterine fibroids. The patient was seen by hematology five months later and hemoglobin electrophoresis was normal so she was advised to follow-up in six months. Subsequently, the patient had another CT scan of the pelvis to follow up on the right buttock fluid collection, as she still had pain and an open wound, and the scan revealed moderate bilateral gluteus maximus muscular atrophy. Full access to the images of this CT scan was also not available to us. The patient was referred to physical medicine and rehabilitation for possible physical therapy and pain management. The patient then relocated to Connecticut and established care with a primary care physician through Nuvance Health.

The patient complained of ongoing nausea and epigastric discomfort after a COVID-19 infection, initially diagnosed over six months prior. This infection had initially been treated supportively with over-the-counter cough medications, but the patient later tried several courses of antibiotics without total resolution of symptoms. She was diagnosed with a long COVID-19 infection four months after her initial COVID-19 diagnosis and complained of brain fog, chronic fatigue, anxiety, and loss of interest in addition to the aforementioned gastrointestinal symptoms.

**Figure 1:**
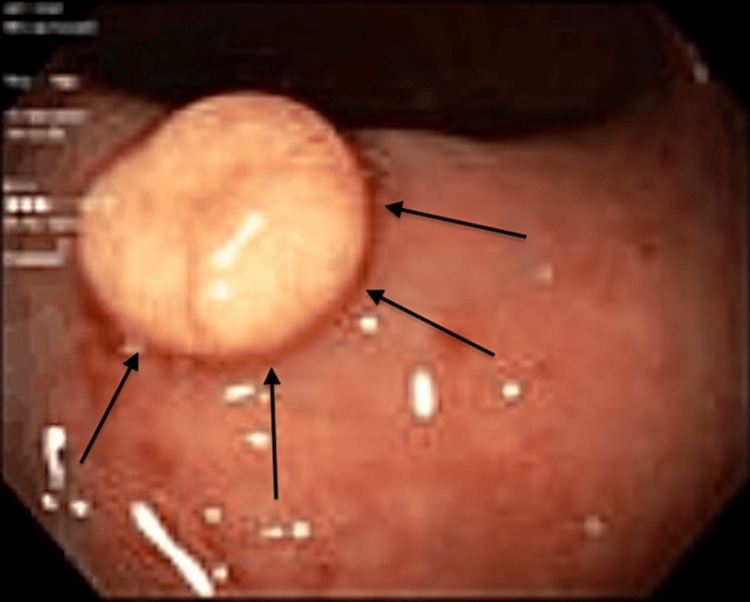
Gross image of the 10mm rectal tumor captured on colonoscopy.

**Figure 2:**
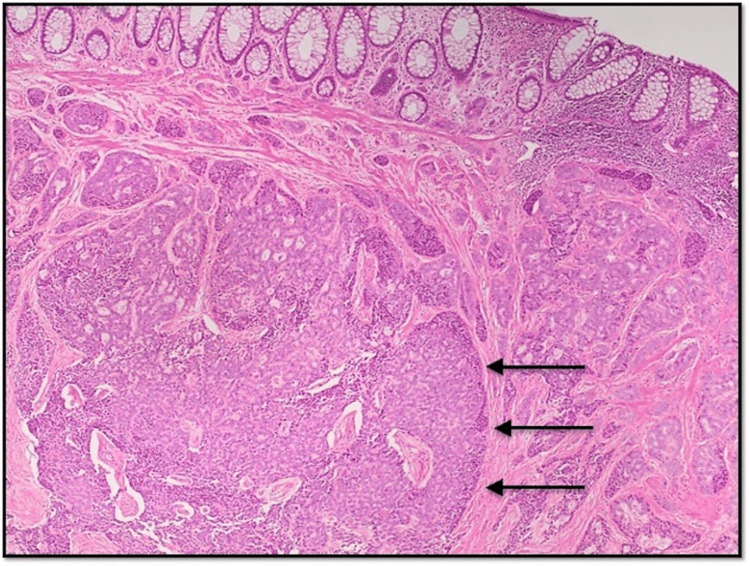
Photomicrograph at 40x magnification (H&E) shows uniform population of tumor cells in lamina propria arranged in nested, organoid, and pseudo-rosette patterns.

**Figure 3:**
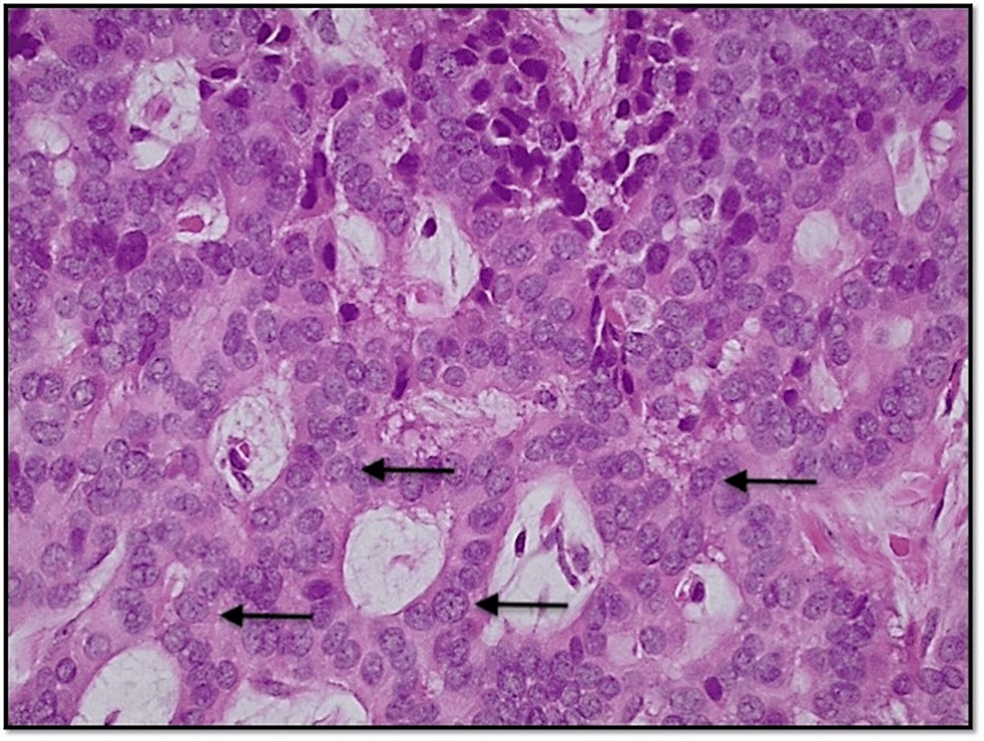
Photomicrograph at 400x magnification (H&E) shows polygonal-shaped cells with salt and pepper chromatin, inconspicuous nucleoli, moderate eosinophilic cytoplasm, rare mitotic figures and no necrosis.

**Figure 4:**
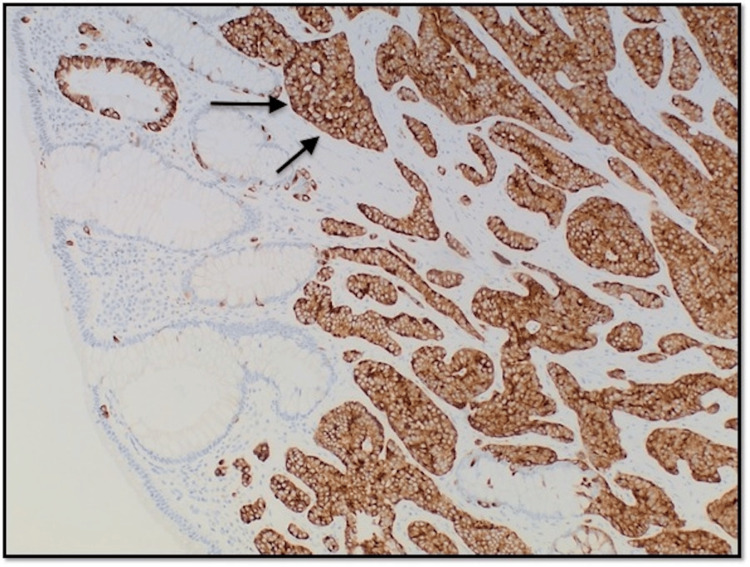
Photomicrograph at 100x magnification of immunohistochemical stain (Synaptophysin) showing lesional cells with positive uptake.

**Figure 5:**
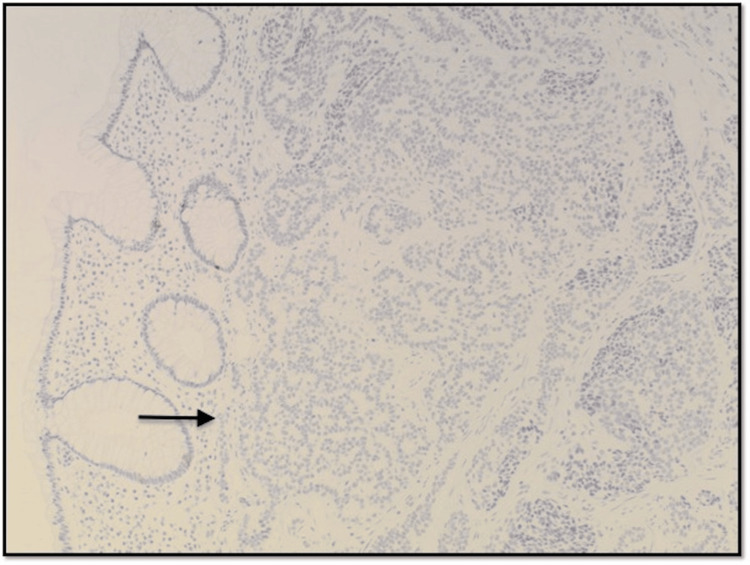
Photomicrograph at 100x magnification of immunohistochemical stain (Chromogranin) showing lesional cells with negative uptake.

**Figure 6:**
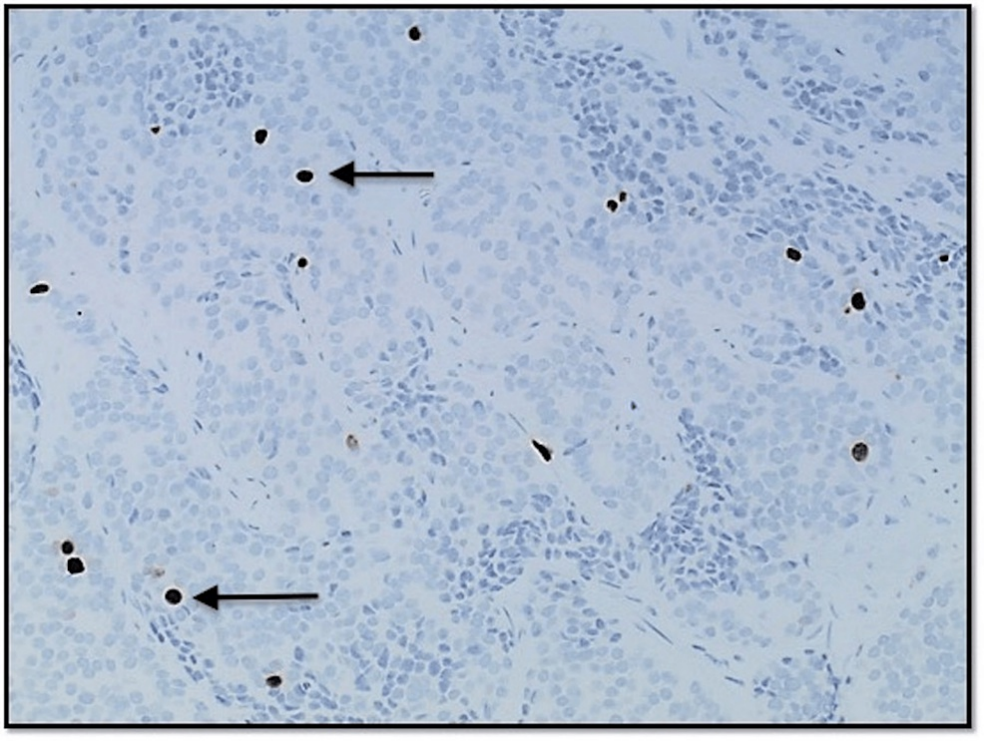
Photomicrograph at 400x magnification of immunohistochemical stain (Ki-67) showing positive nuclear staining in 1.5% of cells.

She was seen by gastroenterology shortly thereafter and was scheduled for an upper endoscopy and screening colonoscopy, for which she had been due for a year prior but had been unable to complete due to the post-surgical complications described above and initial reluctance to undergo a colonoscopy. After extensive counseling on the benefits of screening colonoscopies by her primary care physician, the patient underwent the procedure at Danbury Hospital. A colonoscopy revealed a 10mm polyp in the rectum (Figure 1), which pathology confirmed was a well-differentiated grade 1 carcinoid tumor (Figures 2-6). The patient was urgently referred to colorectal surgery due to concern for submucosal involvement of the tumor.

**Figure 7:**
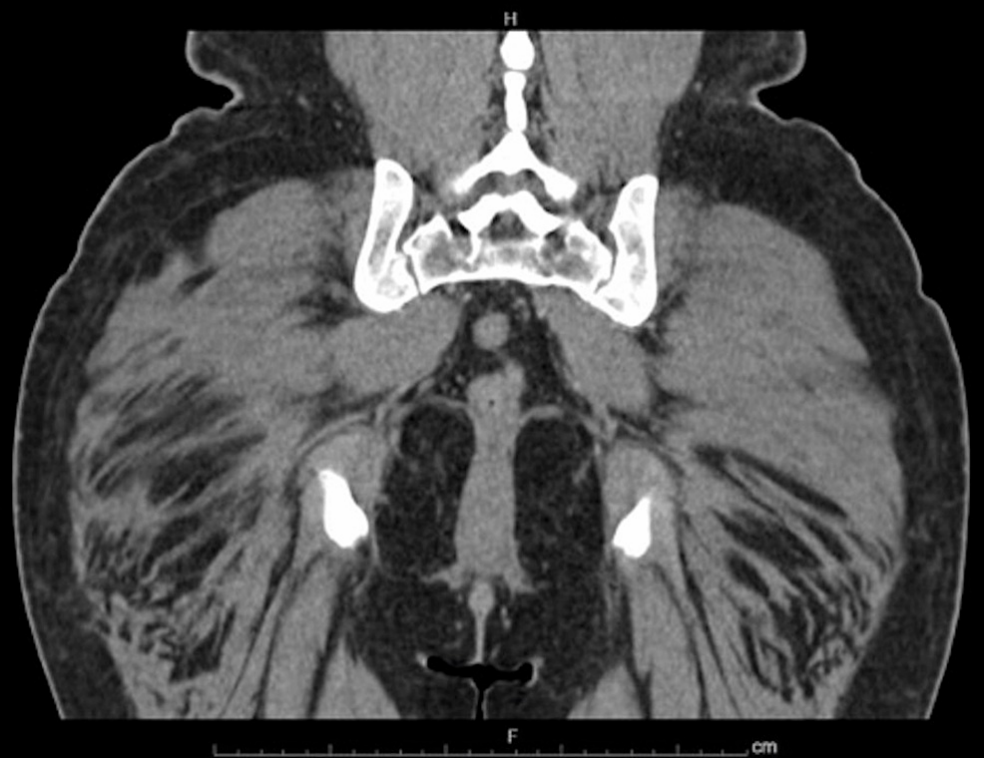
Transverse CT image of the pelvis without any sign of tumor burden or metastatic disease.

**Figure 8:**
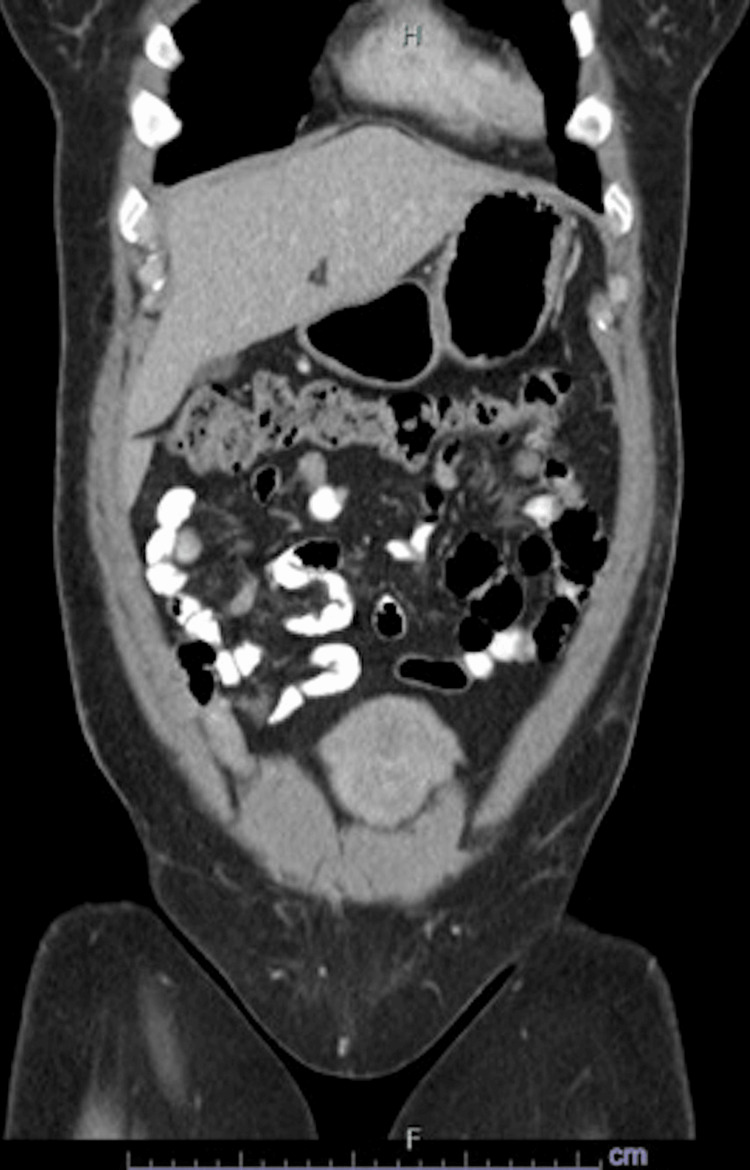
Coronal CT image of the abdomen and pelvis without any sign of tumor burden or metastatic disease (more anterior).

**Figure 9:**
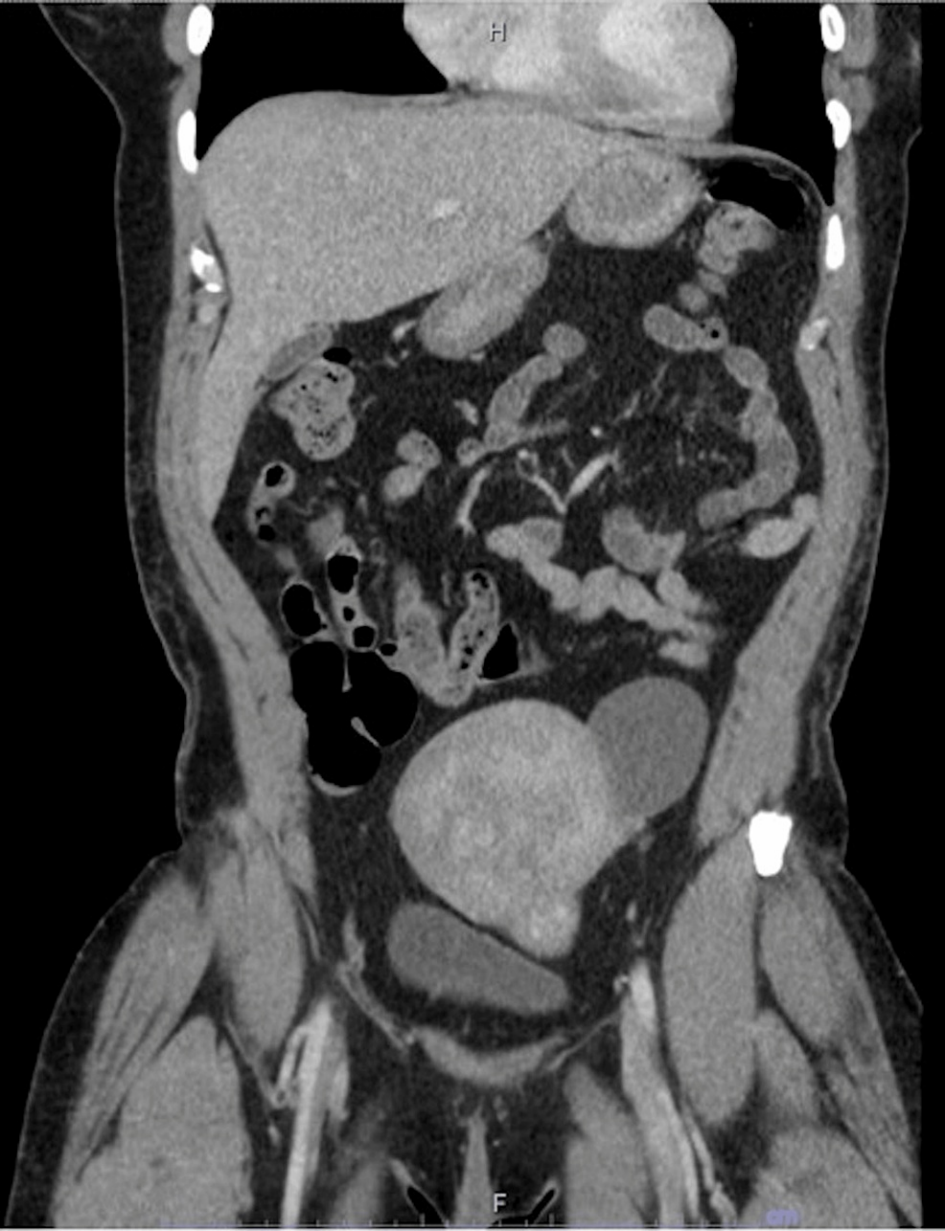
Coronal CT image of the abdomen and pelvis without any sign of tumor burden or metastatic disease (more posterior).

The patient was seen by colorectal surgery and complained of symptoms of anxiety, palpitations, and excessive sweating at night. She denied frequent urination, unintentional weight loss, and shortness of breath. Chromogranin-A level was normal at 22ng/mL. She was briefly hospitalized thereafter due to nausea, diarrhea, lightheadedness, and abdominal pain that had been worsening since her colonoscopy. Still, the hospital work-up did not detect any significant abnormalities, and a CT scan of the abdomen and pelvis did not reveal any signs of metastatic disease (Figures 7-9).

The patient underwent a transanal resection of the rectal carcinoid tumor the following month and the specimen was negative for residual neoplasia. She was seen in the office approximately one week later and reported improvement in symptoms, with only mild right buttock pain and a small amount of post-surgical hematochezia. The patient is currently doing well and reports significant improvement in all her symptoms and has healed well from her surgery.

## Discussion

The incidence of neuroendocrine neoplasms is rising globally, possibly due to increased healthcare utilization and/or improved diagnostic modalities [5]. The largest percentage of these tumors are found in the bronchopulmonary area, followed by the ileum, and then the rectum. Only about 16% of neuroendocrine tumors are found in the rectum [6]. Further, carcinoid tumors make up only about half a percent of all cancers [7] and of all rectal neoplasms, only 1%-2% are carcinoid tumors [8]. Thus, rectal carcinoid tumors are very rare to find in patients, and approximately just half of rectal carcinoid tumors are diagnosed incidentally [9], which was the case in the described patient. While many patients will have a good prognosis, these tumors do have the ability to invade and metastasize, so some patients will require radical surgery and therapy [10]. Carcinoid tumor metastasis can cause complications such as carcinoid syndrome; changes in blood pressure, heart rate, and glucose control; carcinoid heart disease; and in some cases, tissue fibrosis [11]. Clearly, these changes can impact other chronic conditions that patients may have. As the described patient already had hypertension and diabetes, it was fortunate that her carcinoid tumor was discovered prior to metastasis before her other chronic conditions were significantly affected.

When carcinoid tumors do require treatment in addition to surgery, determining tumor characteristics that can guide therapeutic approaches and patient counseling is critical. However, this is often difficult for such a rare condition, so efforts to formally standardize findings, protocols, and recommendations are ongoing [2]. Further, pre-existing co-morbid conditions that can be affected by treatments and the tumor itself, such as diabetes and hypertension in the described patient, must be considered.

As the described patient has diabetes, her primary care physician did consider the possible effects of her carcinoid tumor in managing her diabetes treatment regimen. The impact of pre-existing diabetes on the prognosis of carcinoid tumors has been investigated, but the evidence is conflicting. One study suggested that patients with diabetes were more likely to have metastatic disease at diagnosis [12]; however, a more recent study did not find this association [13]. Another study suggested that preoperative hyperglycemia, not diabetes, was associated with increased tumor size and metastatic disease [14]. If diabetes and/or hyperglycemia are associated with more severe disease and a higher risk of metastasis at diagnosis, then it would be expected that these patients would have poorer outcomes than patients without these conditions, but this has not been demonstrated in the literature [15,16]. The conflicting results of these studies highlight the need for more research to reach a more definitive consensus.

Carcinoid tumors themselves can also impact glycemic control in patients with diabetes. Granin glycoproteins belong to a family of nine acidic proteins that are produced by neuroendocrine tumors, including carcinoid tumors. They function to sort peptides and proteins into secretory granules and their cleavage products are also involved in extracellular regulation of many processes [17]. One such granin protein, chromogranin A, has been found in higher quantities in patients with worse glycemic control. Conversely, another study found that type 2 diabetes patients often had chromogranin A levels in the normal range [17]. Thus, the relationship between this granin and type 2 diabetes also requires further investigation. In type 1 diabetes, chromogranin A has been found to be elevated in 20% of patients and elevated chromogranin A was associated with an increased amount of glycated hemoglobin [17]. Thus, the association between higher chromogranin A with poorer glycemic control in type 1 diabetes has been fairly well established.

Carcinoid tumor treatments may impact glycemic control as well. The ideal scenario is total surgical resection of the tumor and local lymph nodes yielding a complete cure. Interestingly, tumors of the appendix and the rectum, as the described patient had, have the best prognosis due to earlier presentation, and often are able to be entirely resected, as demonstrated in the described case. However, for more advanced diseases, biotherapy using somatostatin analogs may be used [18]. Somatostatin analogs may inhibit the secretion of insulin and glucagon, thus impacting glycemic balance [3]. Everolimus has also been used as a therapeutic agent both alone and in combination with the somatostatin analog, octreotide, and has been associated with hyperglycemia [19]. This highlights the importance of closely monitoring blood glucose levels and adjusting medication regimens appropriately in patients on certain carcinoid tumor treatments to ensure that they remain under good glycemic control.

It is also important to note that in 2021, the US Preventive Services Task Force issued a change to the recommended age that patients should consider beginning colorectal cancer screenings, from 50 to 45 years of age [20]. As the described patient was 46 years old, had this recommendation not been changed and she not been counseled to get a colonoscopy when she did, her rectal carcinoid tumor may not have been found incidentally, but rather after more significant invasion and metastasis. Thus, this case not only highlights the importance of recognizing the impact of carcinoid tumors and their treatments on pre-existing health conditions as well as standardizing guidelines for treatment but also emphasizes the importance of considering current recommended screening guidelines, which are frequently updated to optimize patient care.

Fortunately, the described patient was able to achieve a complete cure with surgical resection of the carcinoid tumor and did well thereafter. She was grateful that the screening colonoscopy was able to identify and treat her condition early. However, in patients with more advanced carcinoid tumors, it is important for providers to monitor pre-existing chronic conditions closely, consider the impact of the tumors and their treatments on such conditions, and ensure that patients are being counseled appropriately on the most recent screening guidelines.

## Conclusions

Carcinoid tumors are quite rare; however, the incidence of neuroendocrine tumors, including gastrointestinal carcinoid tumors, is growing. Thus, it is important to consider the implications of such tumors on pre-existing chronic conditions. Also, more research efforts should be directed towards standardizing the treatment protocol for such tumors, specifically rectal carcinoid tumors, and counseling patients on the importance of screening guidelines. When rectal carcinoid tumors are caught early, complete surgical resection can be curative, however, with more advanced disease, symptoms of the tumor itself as well as necessary treatments can impact other chronic conditions, possibly requiring modifications to a patient’s prior medication regimen. Fortunately, the carcinoid tumor in the described patient was not advanced enough to cause significant alteration in her other chronic conditions, and the patient was grateful that she was counseled on the importance of undergoing the recommended screening so that her condition could be identified and treated early. However, this case is still a great example of how carcinoid tumors can arise asymptomatically, which is why it is important for the astute clinician to counsel on screening recommendations and understand how to manage the whole patient, should a carcinoid tumor arise. This emphasizes the importance of using a team-based approach to ensure that the carcinoid tumor is adequately treated without significantly affecting other conditions so that patients can achieve optimal outcomes for each condition being managed. It also highlights the crucial importance of counseling patients on screening guidelines so that conditions can be caught early to mitigate downstream consequences.
